# The association between green space, socioeconomic deprivation, and COVID-19 infection: a population-based cross-sectional study

**DOI:** 10.3389/ijph.2026.1609558

**Published:** 2026-10-01

**Authors:** Ruoyu Wang, Lynsey Patterson, Aideen Maguire, Ruth F. Hunter

**Affiliations:** 1 Centre for Public Health, Faculty of Medicine, Health and Life Sciences, Queen’s University Belfast, Belfast, United Kingdom; 2 Campus de Campolide, Universidade Nova de Lisboa NOVA Information Management School, Lisbon, Portugal; 3 Institute of Public Health and Wellbeing, University of Essex, Colchester, United Kingdom

**Keywords:** COVID-19 infection, cross-sectional analysis, green space, Northern Ireland, socioeconomic deprivation

## Abstract

**Objectives:**

To assess the association between green space, socioeconomic deprivation, and infection with COVID-19 in Northern Ireland (NI).

**Methods:**

This cross-sectional study included all NI individuals who were tested for COVID-19 in 2020 and 2021 (N = 519,005). Analyses were conducted using multilevel logistic regression models. The proportion of green space was measured within neighborhoods in 2020 and expressed as quintiles.

**Results:**

Of the participants, 318,198 (61.31%) had records of testing positive for COVID-19. The results indicated that individuals living in areas with higher woodland cover had lower odds of reporting a positive COVID-19 test result (e.g., fifth quintile: OR = 0.939; 95% CI = 0.894, 0.987). However, individuals living in areas with higher grassland cover had higher odds of reporting a positive COVID-19 test result (e.g., fifth quintile: OR = 1.062; 95% CI = 1.018, 1.108). These associations were stronger in deprived neighborhoods.

**Conclusion:**

Woodland may provide opportunities for safer social contact while attempting to balance the risk of viral transmission and the potential for positive health behaviors, particularly in more socioeconomically deprived areas. The government should work with urban planners and improve the availability of green spaces as part of future pandemic preparedness strategies.

## Introduction

As of 2024, the World Health Organization reported a total of 776 million infected cases of Coronavirus disease (COVID-19) and more than 7 million fatalities [1]. An emerging line of research arising from the pandemic examines the association between neighborhood physical environments and their impact on both infectious disease transmission and the effectiveness of prevention measures (e.g., social distancing) [2].

Green spaces, an important element of a neighborhood’s physical environment, have attracted significant attention regarding their usage and health benefits during the pandemic [3]. There is evidence that green space exposure supports immunological homeostasis, which may be important for the prevention of diseases [4]. However, evidence of the association between green space and COVID-19 infection is inconsistent [5–7]. On the one hand, some studies have found that more green space is associated with fewer cases of COVID-19 [5–9]. For example, an ecological study in the United States indicated that a greater proportion of forest land is associated with a lower rate of infection with the virus [9]. On the other hand, other ecological studies have suggested that there is a positive association between green space (e.g., proportion of overall green space) and COVID-19 infection [6, 10, 11]. In this regard, one study found that areas with more green space had a higher risk of infection transmission in London, UK [11]. Also, there is evidence that trees and grasses may have heterogeneous effects on health, as the presence of trees has been associated with increased physical activity, while the presence of grass has been associated with decreased physical activity [12].

There also exist socioeconomic inequalities in terms of COVID-19 infection, which means that individuals with lower socioeconomic status (SES) or who live in socioeconomically deprived neighborhoods are more likely to be infected with the virus [13, 14]. One possible solution to mitigate these inequalities could be through interventions in neighborhood physical environments, which is cost-effective and can target a large population [2]. For example, the “equigenesis” theory suggests that socioeconomically deprived groups may be more influenced by public amenities (e.g., green spaces) due to a lack of other resources [15–17]. This indicates that green spaces could narrow socioeconomic inequities in chronic diseases [18]. However, since the majority of green space/COVID-related studies are based on ecological study designs, individual-level evidence of the “equigenesis” of green space on the spread of COVID-19 infection is still limited and needs to be further explored using population-based data [7]. This could help us understand whether adequate access to and promotion of neighborhood green spaces could be a possible solution to reducing socioeconomic inequalities at the pandemic level (e.g., viral infection rates) and reducing transmission risk through safe social contact while also providing a space to be physically active.

The current study aims to contribute to the existing literature in the following ways. First, previous studies on the health effects of green spaces were primarily focused on chronic diseases, while scant attention has been paid to infectious diseases [4, 7, 19]. The majority of existing studies on COVID-19 infection were based on aggregated data and had an ecological study design, the findings of which may not be valid for individuals [7]. Therefore, this study aims to understand the associations between green space and COVID-19 infection at the individual level. Second, the “equigenesis” theory has rarely been examined in the context of COVID-19 infection; therefore, we aim to investigate whether the association between green space and COVID-19 infection is more pronounced in individuals with lower SES or those living in deprived neighborhoods, while still contributing to the literature on equigenesis.

## Methods

### Study design and samples

A population-based, data-linkage study design was used to investigate the association between green space, socioeconomic deprivation, and COVID-19 infection in Northern Ireland (NI) during the first two years of the pandemic (2020 and 2021, due to data availability) using the data from the National Health Application and Infrastructure Services (NHAIS) system hosted by the Honest Broker Service (HBS) [20, 21]. This system contains information on all residents registered with a General Practice (GP) in NI [20, 21]. NI has a “free at the point of service” national health service, and so nearly the entire population is registered with a GP (>98%) [20, 21]. The NHAIS data include residents’ basic demographic information and health and care numbers (HCNs), which can be further used to link to the COVID-19 testing data [20, 21]. Data on rateable house value were obtained from Land and Property Services, and area-level deprivation and urbanity data were obtained from the NI Multiple Deprivation Measure and Review of the Statistical Classification and Delineation of Settlements [22, 23]. The Health and Social Care Northern Ireland (HSCNI) Business Services Organisation (BSO), through HBS, performed the linkage and provided researcher access to de-identified datasets within a Trusted Research Environment. This study was approved by the HSCNI HBS Governance Board (project 083) and the Research Governance and Ethics at Queen’s University Belfast.

According to NI’s Department of Health, the cumulative number of COVID-19 cases was 72,834 in 2020 and 402,069 in 2021 [24]. The initial number of records about individuals tested for COVID-19 (a single person can have multiple test records) is 6,437,770 from 29 March 2020 to 31 December 2021. We only included individuals who fulfilled the following eligibility criteria: alive and resident in NI from 2020 to 2021, not in an institution (e.g., living in a care home or prison), and who had both complete demographics (see covariates below) and COVID-19 testing information. Samples were limited to subjects living in the residential community, since living environments and associated restrictions may differ between residential communities and those living in institutions in NI [20, 21]. The flowchart of the cohort selection for this study is shown in Supplementary Figure S1, and the final sample size was 519,005 unique individuals.

### COVID-19 testing data

The SARS-CoV-2 testing in NI began on 7 February 2020 and was managed by the Regional Virus Laboratory, Belfast Health and Social Care Trust. The testing data in this study included both Pillar 1 testing [25] and Pillar 2 testing data [26] in 2020 and 2021, which were then linked to NHAIS samples. Pillar 1 testing is provided by the Laboratory Information Systems in NI hospitals, while Pillar 2 testing is provided by the National Health Service (NHS) Digital. Since there may be multiple tests for a single person, we treated COVID-19 testing data as a binary variable (1 = the resident tested positive in any of the records in any of the years; 0 = the resident tested negative in all records in both 2020 and 2021). We excluded records with missing or undefined (e.g., unknown) information.

### Green space

The availability of green space was measured using the Land Cover Map 2020 vector data [27]. In the UK, Wales recorded the highest average share of green space (45%), with lower values observed in Northern Ireland (24%), England (21%), and Scotland (16%) [16]. The Land Cover Map was provided by the UK Centre for Ecology & Hydrology based on satellite images of the UK. It includes land use information on a variety of classes [27], and green space refers to grassland (improved grassland, neutral grassland, calcareous grassland, acid grassland, and heather grassland) and woodland (broadleaved woodland and coniferous woodland). Since existing studies have found that there are heterogeneous health effects of grassland and woodland [28], we have distinguished the effects between them in the analysis. Residents’ addresses in January 2020 were used to match their areas of residence to Super Output Areas (SOAs), which are the primary administrative units (containing between 2,000 and 6,000 households) in NI. These are similar to the Lower Layer Super Output Areas (LSOAs) in England and are typically used to assign area-level factors since they could reflect the daily scope of activities and are the finest geographical unit for digital health records in the UK [20, 21]. The proportion of grassland and woodland was calculated within the SOAs. These variables were operated as quintiles (Q1 being the least green to Q5 being the most green) to test the non-linear effects of green space and to meet the HBS’s privacy protection regulations.

### Covariates

The NHAIS database includes sociodemographic information on individuals. We controlled for sex, age, Area level Multiple Deprivation Measure (MDM) income domain [22], urbanity [23], and capital values of the house. The Area level Multiple Deprivation Measure (MDM) income domain (Q1 being the most deprived, Q5 being the least deprived) is a composite index that measures the level of economic deprivation in communities [22]. Urbanity was measured by settlement band, which classifies communities into different bands (A-H) based on population size [23]. Based on NISRA et al. [23], bands A-D are urban (population greater than 5,000), and bands E-H are rural. Following previous studies [20, 21], we categorized capital values of the house into five grades (G1: ≤ £99,999; G2: £100,000-£199,999; G3: £200,000-£299,999; G4: £300,000-£399,999; G5: ≥ £400,000). Summary statistics of the variables are presented in Table 1.

**TABLE 1:** Statistical summary of variables (Northern Ireland, 2020–2021).

Variables	Number of observations (%)	Mean (SD)
Covariates		
Sex		
Male resident	237,576 (45.8)	​
Female resident	281,429 (54.2)	​
Age		
<18 years	107,382 (20.7)	​
18–29 years	105,552 (20.3)	​
30–49 years	168,685 (32.5)	​
50–69 years	105,560 (20.3)	​
>69 years	31,826 (6.1)	​
Urbanity		
Urban area	280,396 (54.0)	​
Rural area	238,609 (46.0)	​
SES indicators		
Northern Ireland multiple deprivation measure (MDM) income domain		
1st quintile (Q1: Most deprived)	115,871 (22.3)	​
2nd quintile (Q2)	113,074 (21.8)	​
3rd quintile (Q3)	110,356 (21.3)	​
4th quintile (Q4)	103,401 (19.9)	​
5th quintile (Q5: Least deprived)	76,303 (14.7)	​
Capital value of housing (capital)		
1st grade (G1: ≤£99,999 [least expensive])	206,783 (39.8)	​
2nd grade (G2: £100,000-£199,999)	237,069 (45.7)	​
3rd grade (G3: £200,000-£299,999)	59,178 (11.4)	​
4th grade (G4: £300,000-£399,999)	11,171 (2.2)	​
5th grade (G5: ≥£400,000 [most expensive])	4,804 (0.9)	​
Green space	​	0.346 (0.360)
Proportion of grassland (grass)		
1st quintile (Q1: Least green)	​	0.001 (0.001)
2nd quintile (Q2)	​	0.008 (0.012)
3rd quintile (Q3)	​	0.150 (0.080)
4th quintile (Q4)	​	0.528 (0.115)
5th quintile (Q5: Most green)	​	0.829 (0.058)
Proportion of woodland (Wood)		
1st quintile (Q1: Least green)	​	0.000 (0.001)
2nd quintile (Q2)	​	0.001 (0.005)
3rd quintile (Q3)	​	0.018 (0.009)
4th quintile (Q4)	​	0.053 (0.012)
5th quintile (Q5: Most green)	​	0.145 (0.076)
Outcome		
COVID-19 test		
Positive	318,198 (61.3)	​
Negative	200,807 (38.7)	​

SD, standard deviation.

### Statistical analysis

To identify the association between green space and the likelihood of being infected with COVID-19, we used multilevel logistic regression models to ensure model convergence and stable maximum likelihood estimates (e.g., a small number of cases in a few stratifications) across a series of stratified analyses [29]. Since some residents were clustered within the same SOA across NI, we used a two-level random intercept model to account for variation in intercepts across SOAs. The Variance inflation factor test (VIF = 1.79) indicated that there was no multicollinearity among the predictors.

First, for the baseline model, we estimated the association between green space and COVID-19 infection without controlling for covariates (Model 1). Second, we added all covariates to Model 1 to set up the adjusted model (Model 2). Additional analyses using the log-binomial or Poisson regression indicate that the results generally remained consistent (Supplementary Figure S2).

The following additional sensitivity tests were performed to examine the robustness of our findings. Since the Area Level Multiple Deprivation Measure is a composite index and the income domain may only reflect the area-level socioeconomic status [20, 21], we used the overall Area Level Multiple Deprivation Measure and re-ran the fully adjusted model (Model 3). We also used the 2019 Land Cover Map to measure green space availability (Model 4). This helped us test whether our findings are sensitive to the lagged effect of green space. We removed residents who had moved from one SOA to another SOA between 2020 and 2022 and re-ran the fully adjusted model (Model 5). Residents with a relocation history may have been influenced by other unobserved individual or environmental factors [30].

The COVID vaccination was first offered to all care home residents and health and social care workers in December 2020, followed by those aged 50 years and over and those aged 18–49 years with underlying health conditions in January 2021. Due to data availability at the time of the original data request, we were unable to obtain access to the COVID-19 vaccination data. Therefore, we added sensitivity analyses using 2020 (before vaccination was offered) and 2021 COVID-19 testing data, respectively (Supplementary Figure S3: Models S1 and S2). Despite some differences in magnitude, the results generally remained consistent between 2020 and 2021. Additionally, the influence of environmental factors should be relatively long-term, so we primarily focused on interpreting results from aggregated samples (2020 and 2021), which could provide more robust and reliable results.

Finally, to test whether the association between green space and the risk of contracting COVID-19 varied across different demographic and socioeconomic groups, we stratified the analysis by sex (male subjects vs. female subjects), age (≤17 vs. 18–29 vs. 30–49 vs. 50–69 vs. ≥70 years), Area level Multiple Deprivation Measure (Q1 to Q5), urbanity (urban vs. rural), and capital values of the house (G1 to G5). The effect modifications of socioeconomic status and demographics in the association between green space and COVID-19 infection were tested by the interaction terms between modifiers and green space. The results are presented as odds ratios (ORs) and 95% confidence intervals (CIs) using Stata v18 (College Station, Texas, USA).

## Results

Table 1 shows the characteristics of the cohort (n = 519,005) of all individuals in NI who had a recorded COVID-19 test result in 2020–2021. The average age of the cohort was 37 years, ∼54% of the subjects were women, and ∼54% lived in urban areas. There were 318,198 (61%) positive COVID-19 test results during the study period.

Figure 1 and Supplementary Table S1 present the multivariate associations between green space, SES, and the odds of a positive COVID-19 test result (Model 1 and Model 2). In the adjusted model (Model 2), individuals living in quintiles 3 (OR = 0.954; 95% CI = 0.929, 0.980), 4 (OR = 0.929; 95% CI = 0.903,0.956), and 5 (OR = 0.896; 95% CI = 0.869, 0.924) of MDM had lower odds of reporting a positive COVID-19 test result relative to those in Q1. Also, individuals whose homes fell within the second capital-value grade (OR = 1.031; 95% CI = 1.017, 1.045) had higher odds of reporting a positive COVID-19 test result, while those living in the fifth capital-value grade (OR = 0.904; 95% CI = 0.847, 0.964) had lower odds of reporting a positive COVID-19 test result.

**FIGURE 1:**
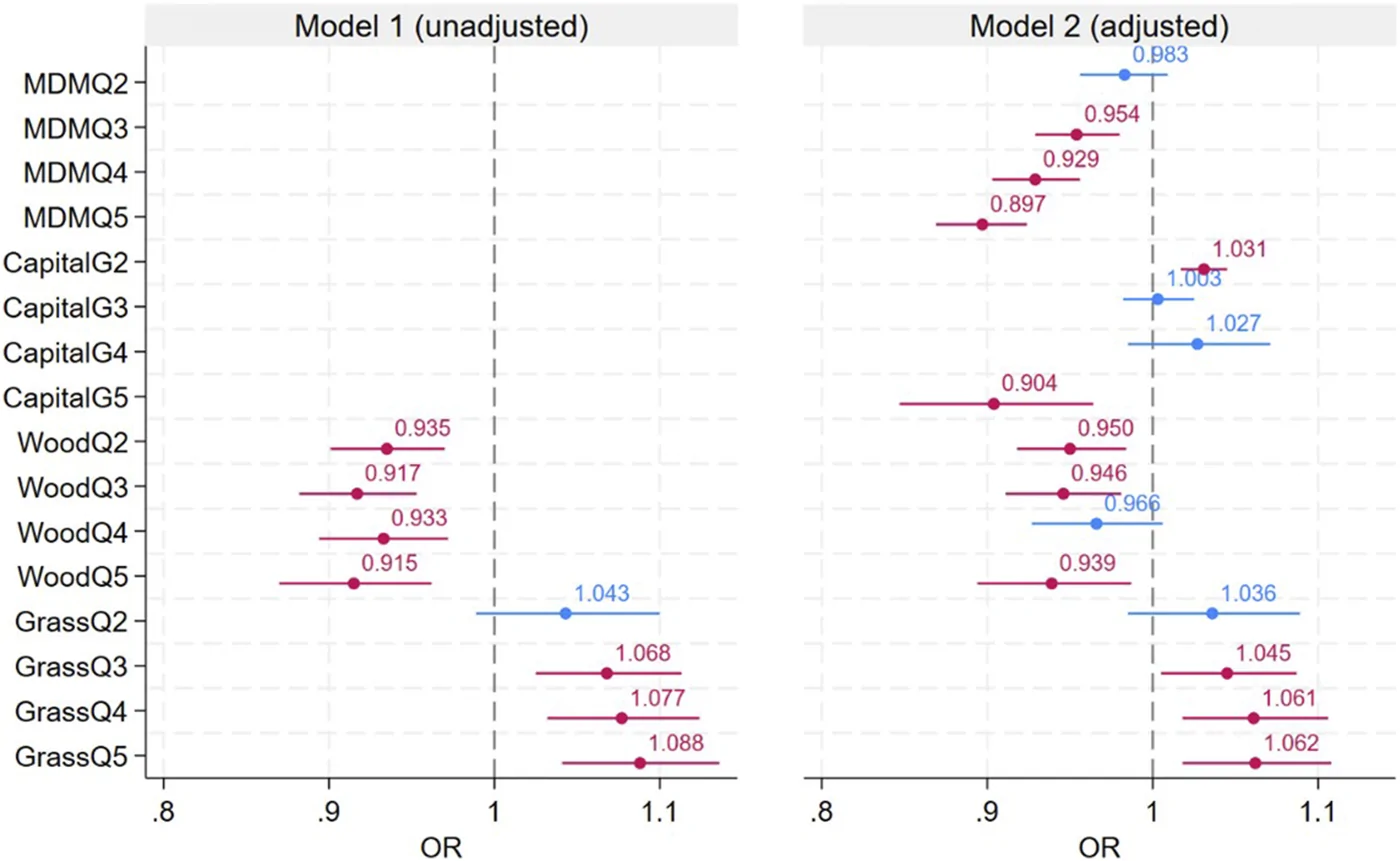
Associations between green space, socioeconomic deprivation, and the odds of testing positive for COVID-19 (Northern Ireland, 2020–2021). Note: Adjusted models were controlled for sex, age, and urbanity. Error bars indicate 95% CIs. Red bars indicate p < 0.05. Error bars indicate 95% CIs. OR, odds ratio; MDM, Northern Ireland Multiple Deprivation Measure income domain; Capital, capital value of housing; Wood, proportion of woodland; Grass, proportion of grassland; Q, quintile; G, grade.

Also, individuals living in the second (OR = 0.950; 95% CI = 0.918, 0.984), third (OR = 0.946; 95% CI = 0.911, 0.981), and fifth (OR = 0.939; 95% CI = 0.894,0.987) quintiles of woodland cover had lower odds of reporting a positive COVID-19 test result relative to the first quintile. However, those living in the third (OR = 1.045; 95% CI = 1.005,1.087), fourth (OR = 1.061; 95% CI = 1.018, 1.106), and fifth (OR = 1.062; 95% CI = 1.018, 1.108) quintiles of grassland cover had higher odds of reporting a positive COVID-19 test result relative to those in the first quintile.

Figure 2 and Supplementary Table S2 summarize the results of the sensitivity analyses examining the associations between green space, SES, and the odds of testing positive for COVID-19. Despite some differences in magnitude, the associations between green space, SES, and the odds of reporting a positive COVID-19 test result remained similar across all models.

**FIGURE 2:**
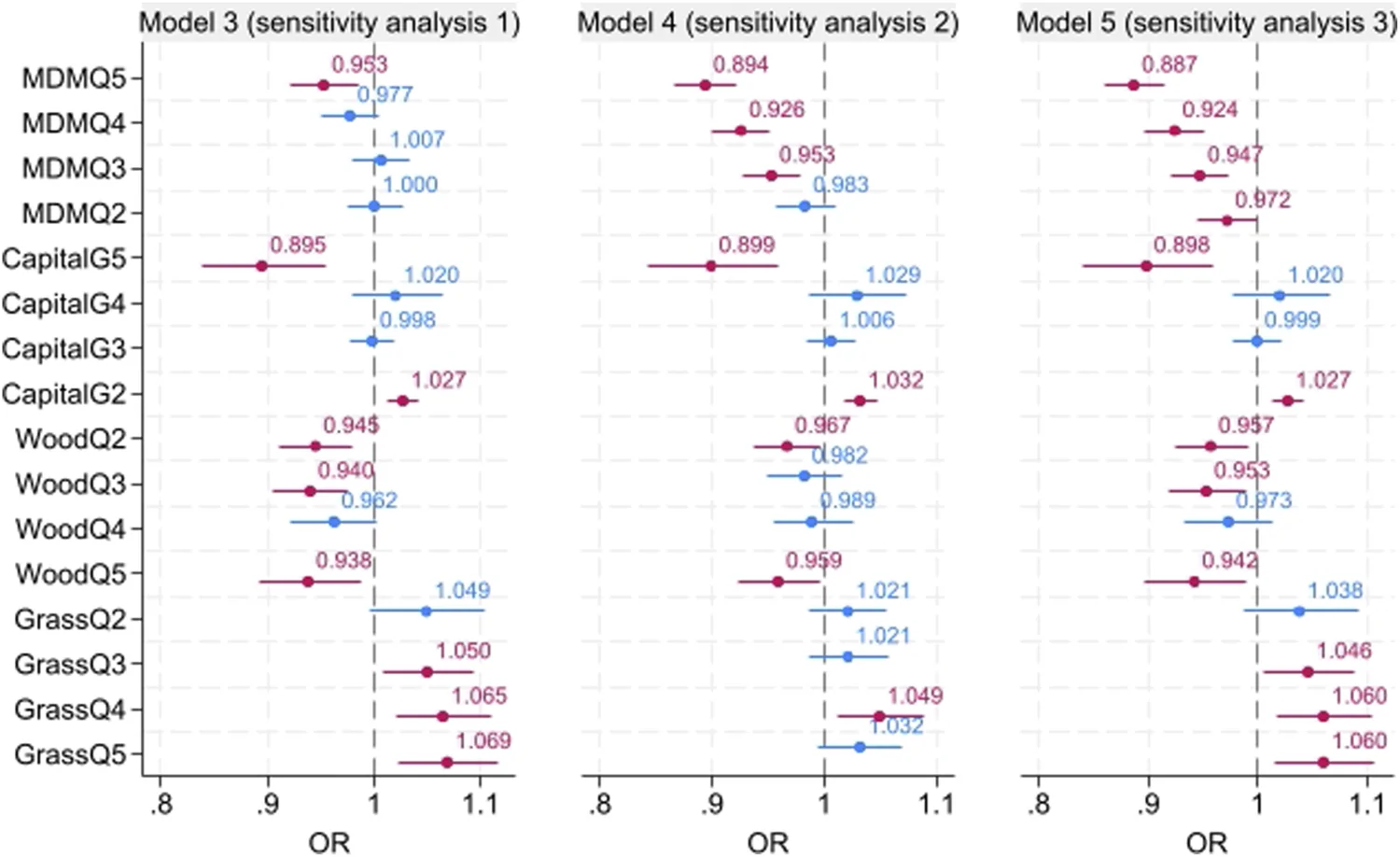
Sensitivity analysis of the associations between green space, socioeconomic deprivation, and the odds of testing positive for COVID-19 (Northern Ireland, 2020–2021). Note: Error bars indicate 95% CIs. Red bars indicate p < 0.05. OR, odds ratio; MDM, Northern Ireland Multiple Deprivation Measure income domain; Capital, capital value of housing; Wood, proportion of woodland; Grass, proportion of grassland; Q, quintile; G, grade.

Figure 3 and Supplementary Table S3 present a stratified analysis of the associations between green space, SES, and the odds of a positive COVID-19 test result. First, the effects of MDM and the capital value of housing were similar for men and women. The effect of woodland cover was more significant in men, while the effect of grassland was more significant in women. Second, although the impact of the capital value of housing was similar across different age groups, a less significant MDM-odds of reporting a positive COVID-19 test association was not statistically significant in minors (<18 years). Third, the association between MDM and the odds of reporting a positive COVID-19 test result was similar in urban and rural areas. However, the association between the capital value of housing and the odds of reporting a positive COVID-19 test result was negative in urban areas, but such an association was positive in rural areas. Additionally, the impact of green space (grassland cover and woodland cover) was more significant in rural areas than in urban areas.

**FIGURE 3:**
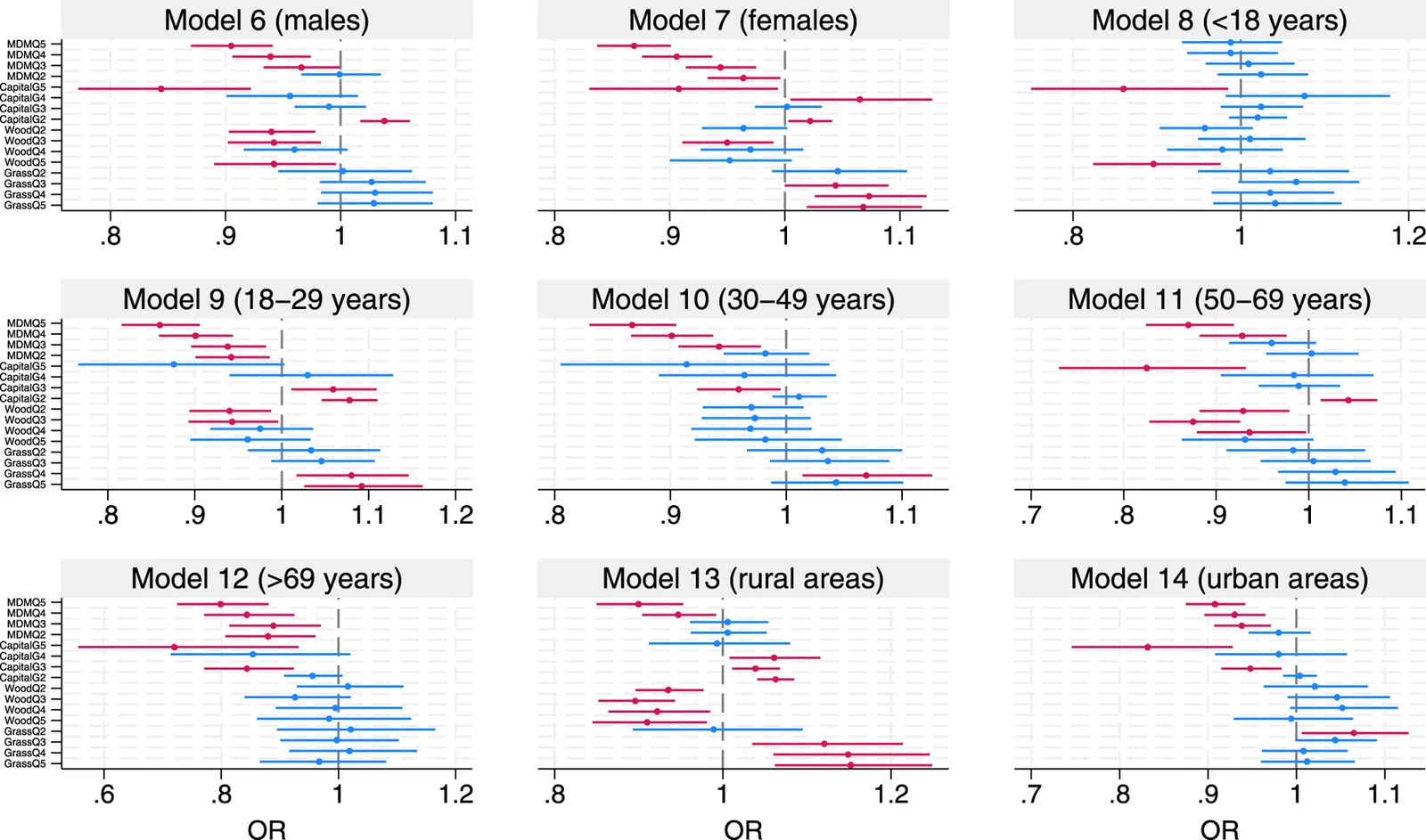
Stratified analysis of the associations between green space, socioeconomic deprivation, and the odds of a positive COVID-19 test (Northern Ireland, 2020–2021). Note: Models were controlled for sex, age, and urbanity. Error bars indicate 95% CIs. Red bars indicate p < 0.05. The p-values for interactions are presented in Supplementary Tables S2, S5. OR, odds ratio; MDM, Northern Ireland Multiple Deprivation Measure income domain; Capital, capital value of housing; Wood = proportion of woodland; Grass, proportion of grassland; Q, quintile; G, grade.

Figure 4, Table 2 and Supplementary Table S4 show the association between green space and the odds of reporting a positive COVID-19 test result, stratified by SES. The results indicate that the effect of green space (grassland cover and woodland cover) was more significant and larger in the lower quintiles of MDM and the capital value of housing. For example, as for individuals living in neighborhoods with MDM Q1, higher quintiles of the proportion of woodland cover were associated with lower odds of reporting a positive COVID-19 test, relative to Q1. However, such an effect was not observed in individuals living in neighborhoods with MDM Q5.

**FIGURE 4:**
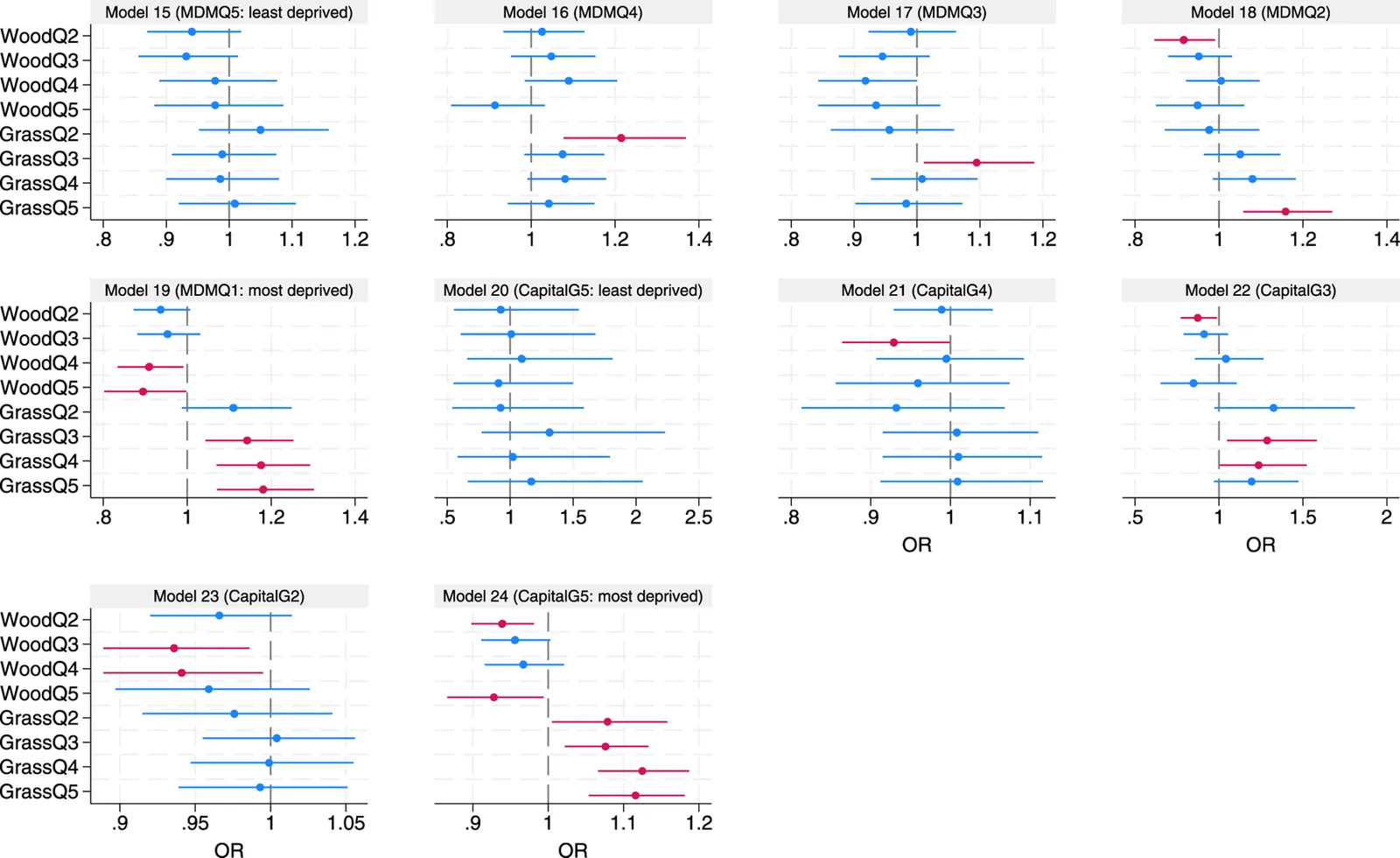
The association between green space and the odds of testing positive for COVID-19 (stratified by SES) (Northern Ireland, 2020–2021). Note: Models were controlled for sex, age, and urbanity. Error bars indicate 95% CIs. Red bars indicate p < 0.05. The p-values for interactions are presented in Table 2. OR, odds ratio; MDM, Northern Ireland Multiple Deprivation Measure income domain; Capital, capital value of housing; Wood, proportion of woodland; Grass, proportion of grassland; Q, quintile; G, grade.

**TABLE 2:** Effect of sociodemographic modifications on the association between green space and the odds of testing positive for COVID-19 (Northern Ireland, 2020–2021).

Variable	P_interaction_				
​	Sex	Age	Urbanity	MDM	Capital
WoodQ2 (ref: WoodQ1)	0.215	0.096	0.013	0.111	0.045
WoodQ3	0.800	0.000	0.000	0.301	0.233
WoodQ4	0.817	0.210	0.000	0.000	0.066
WoodQ5	0.080	0.000	0.000	0.001	0.003
GrassQ2 (ref: GrassQ1)	0.021	0.014	0.013	0.006	0.121
GrassQ3	0.647	0.193	0.000	0.045	0.038
GrassQ4	0.191	0.563	0.000	0.011	0.000
GrassQ5	0.251	0.053	0.000	0.003	0.001

MDM, Northern Ireland Multiple Deprivation Measure income domain; Capital, capital value of housing; Wood, proportion of woodland; Grass, proportion of grassland; Q, quintile.

## Discussion

The results of this population-based cross-sectional study showed that higher proportions of woodland cover were associated with lower odds of testing positive for COVID-19, while higher proportions of grassland cover were associated with higher odds of a positive COVID-19 test result. Green spaces, such as grassland, could provide more open spaces during a pandemic and may encourage more outdoor activities and social interactions, which may in turn lead to a higher risk of COVID-19 infection [5]. Conversely, woodland may provide shade from sunlight and encourage more solitary physical activity, such as walking [31–33]. A consequence of this is that individuals are more likely to move from place to place within woodland, maintaining social distancing and avoiding frequent contact, ultimately reducing the risk of COVID-19 infection. Also, the “hygiene hypothesis” suggests that contact with microbial antigens in green spaces may strengthen people’s immune function [34, 35], which may also partly explain the lower risk of COVID-19 infection in woodland. However, it should be noted that this study does not provide conclusive evidence that woodland and grassland have heterogeneous effects on the spread of COVID-19, since important factors influencing the usage of grassland and woodland were not measured in this study (e.g., population density, urban park usage patterns, recreational clustering, and unmeasured neighborhood characteristics).

Our findings indicate that individuals with a lower capital value of housing or living in more deprived neighborhoods were more likely to test positive for COVID-19. One possible explanation is that the quality of green space in deprived neighborhoods is low (e.g., poorly maintained), so its health benefits may be limited [16, 36]. Also, there are fewer publicly accessible green spaces (e.g., public parks) in deprived neighborhoods; therefore, individuals in these neighborhoods may not be able to take full advantage of existing green spaces (e.g., club-based green spaces) [37]. For example, a study in the UK found that there are fewer green spaces in more deprived neighborhoods compared with less deprived neighborhoods [16]. Another possible explanation is that individuals with a lower SES or living in deprived neighborhoods were usually more financially constrained and more vulnerable during a pandemic [38]. For example, previous studies have revealed that socioeconomically deprived groups were less likely to work from home during the pandemic, thereby increasing their likelihood of coming into contact with someone who was infected [38]. Also, individuals with a lower SES or living in deprived neighborhoods are more likely to have less health-related knowledge and information, so they may have underestimated the importance of public health messaging around strategies for mitigating the risk of contracting COVID-19 during the pandemic [39].

Our evidence also suggests that the association between green space (woodland cover) and a positive test result for COVID-19 infection was stronger in more deprived neighborhoods or groups with a lower capital value of housing, which indicates that socioeconomically deprived groups are more affected by the availability of green space (woodland cover). This also implies that areas with more green space (woodland cover) may have fewer SES inequalities in terms of COVID-19 infection. Public amenities, such as green spaces, are usually freely accessible to all residents; therefore, individuals in socioeconomically deprived groups with fewer health-related resources may rely more on them to fulfill their needs. For example, individuals in socioeconomically deprived groups were less likely to have a private garden, so they were possibly more likely to seek more public green spaces for physical activity during the pandemic, which may have helped strengthen their immunity to combat the virus [34 35]. Additionally, the pandemic caused greater mental distress in individuals from socioeconomically deprived groups [40], and green space, such as woodland, may have exerted a restorative effect on individuals, reducing their levels of stress [41]. Existing studies have suggested that individuals with mental disorders are at a higher risk for contracting COVID‐19 [42], so socioeconomically deprived groups, who are under greater mental stress, were also more vulnerable.

As for other heterogeneous effects of green space and socioeconomic deprivation, the influence of socioeconomic deprivation was similar for men and women. However, the beneficial effect of woodland cover was stronger for men, while the stronger positive association between grassland and the odds of a positive COVID-19 test among women. This may be because men tended to use green space more for physical activity [43], while women tended to use green space more for social contact during the pandemic [44]. In addition, the results show that the effect of socioeconomic deprivation was weaker for minors (<18 years), while the effect of green space was weaker in older adults. Existing studies have found that children and younger adults have better immune function and thereby are more resistant to infection regardless of SES factors [45]. Older adults are more likely to have lower levels of mobility due to functional restrictions, and this was especially true during the pandemic [46]. This may discourage their use of green space. We also found that the effect of green space was stronger in rural areas. One possible explanation is that rural areas are less populated and had fewer restrictions on outdoor activities during the pandemic [47], so individuals could have used green spaces more frequently without worrying about social distancing [48].

### Limitations

This study has some limitations. First, our data were based on a cross-sectional design, meaning that we may not be able to infer the causality between green space, socioeconomic deprivation, and COVID-19 infection in NI. Second, the dataset on the number of individuals tested for COVID-19 was developed in a rapid response to the pandemic, and it included COVID-19 tests using antigen swabs performed at various testing locations. However, the number of cases may still be an underestimation, as there were asymptomatic cases who did not get tested. Third, to protect privacy, green space exposure was measured at the level of the administrative unit rather than at the housing address level. Therefore, this method may not accurately reflect individuals’ daily green space exposure and may result in the Modifiable Areal Unit Problem (MAUP) [49]. For example, the green space exposure quintiles may change if other geographical units were to be used (e.g., electoral wards), and this may have had an influence on the associations between green space and contracting COVID-19. Alternative exposure metrics (e.g., distance-based buffers or accessibility indices) were not considered because we lack accurate housing locations for the participants. Fourth, as our data were based on administrative routine data, there were only a limited number of covariates available. Therefore, we may not have included all important individual-level (e.g., occupational exposure risk and mobility patterns) or institutional-level covariates (e.g., vaccination rollout, testing availability, and lockdown policies) [50]. Because the study period (2020–2021) coincided with multiple changes, the observed associations may have been biased by the independent influence of the above changes. We did explore this possibility through a sensitivity analysis (by excluding 2021 when the vaccine program was introduced), and the results remained consistent. Finally, we lacked information on individuals’ use of green space during the pandemic, so the actual mechanisms linking green space to COVID-19 infection are still unclear and are based on well-known assumptions in the literature.

### Conclusion

Our findings suggest that exposure to green space, especially woodland cover, is associated with lower levels of COVID-19 infection in NI, especially in socioeconomically deprived neighborhoods. Future public health strategies to reduce viral infection rates during a pandemic may benefit from encouraging the use of woodland space rather than avoiding all types of outdoor activities, which is beneficial for other health behaviors, such as physical activity. The government should work with urban planners and improve the availability of green spaces as part of future pandemic preparedness strategies.

## Data Availability

The data used in this study cannot be publicly deposited. Project specific data-sets are created and destroyed in line with the Administrative Data Research Centre Northern Ireland Guidelines. Metadata for each project is available on request. Access to the data sets used can be obtained through application to the Health and Social Care Northern Ireland Business Service Organisation’s Honest Broker Service https://hscbusiness.hscni.net/services/2454.htm.
