# Supplementary material for: The association between green space, socioeconomic deprivation, and COVID-19 infection: a population-based cross-sectional study
Source: Int J Public Health. 2026 Oct 1;71:1609558. doi: 10.3389/ijph.2026.1609558 (PMC13668763; doi:10.3389/ijph.2026.1609558)
Supplement: Supplementary file 1 [file Supplementaryfile1.doc]

**Figure S1:** Flow chart of the study sample selection.

**Figure S2:** The association between green space, SES, and the prevalence of having a positive COVID-19 test in 2020 and 2021, respectively. Adjusted models were controlled for sex, age, and urbanity. Error bars indicate 95% CIs. Red bars indicate p < 0.05. Error bars indicate 95% CIs. PR = prevalence ratios; MDM = Northern Ireland Multiple Deprivation Measure income domain; Capital = capital value of housing; Wood = the proportion of woodland; Grass = the proportion of grassland; Q = quintile; G = grade.

**Figure S3:** The association between green space, SES, and the odds of having a positive COVID-19 test in 2020 and 2021, respectively. Adjusted models were controlled for sex, age, and urbanity. Error bars indicate 95% CIs. Red bars indicate p < 0.05. Error bars indicate 95% CIs. OR = odds ratio; MDM = Northern Ireland Multiple Deprivation Measure income domain; Capital = capital value of housing; Wood = the proportion of woodland; Grass = the proportion of grassland; Q = quintile; G = grade.

Table S1: The association between green space, SES, and the odds of having a positive COVID-19 test.

|  | Unadjusted | | Adjusted | |
| --- | --- | --- | --- | --- |
|  | OR | 95% CI | OR | 95% CI |
| Male (ref: Female) |  |  | 1.179 | 1.165, 1.192 |
| Age:18-29 (ref: Age: <19) |  |  | 0.621 | 0.609, 0.632 |
| Age: 30-49 |  |  | 0.572 | 0.562, 0.581 |
| Age: 50-69 |  |  | 0.435 | 0.427, 0.443 |
| Age: >69 |  |  | 0.287 | 0.280, 0.295 |
| Urban area (ref: Rural area) |  |  | 0.988 | 0.967, 1.009 |
| MDMQ5 (ref: MDMQ1) |  |  | 0.896 | 0.869, 0.924 |
| MDMQ4 |  |  | 0.929 | 0.903, 0.956 |
| MDMQ3 |  |  | 0.954 | 0.929, 0.980 |
| MDMQ2 |  |  | 0.983 | 0.956, 1.009 |
| CapitalG5 (ref: CapitalG1) |  |  | 0.904 | 0.847, 0.964 |
| CapitalG4 |  |  | 1.027 | 0.985, 1.071 |
| CapitalG3 |  |  | 1.003 | 0.982, 1.025 |
| CapitalG2 |  |  | 1.031 | 1.017, 1.045 |
| WoodQ2 (ref: WoodQ1) | 0.935 | 0.901, 0.970 | 0.950 | 0.918, 0.984 |
| WoodQ3 | 0.917 | 0.883, 0.953 | 0.946 | 0.911, 0.981 |
| WoodQ4 | 0.933 | 0.894, 0.972 | 0.966 | 0.927, 1.006 |
| WoodQ5 | 0.915 | 0.870, 0.962 | 0.939 | 0.894, 0.987 |
| GrassQ2 (ref: GrassQ1) | 1.043 | 0.989, 1.100 | 1.036 | 0.985, 1.089 |
| GrassQ3 | 1.068 | 1.025, 1.113 | 1.045 | 1.005, 1.087 |
| GrassQ4 | 1.077 | 1.032, 1.124 | 1.061 | 1.018, 1.106 |
| GrassQ5 | 1.088 | 1.041, 1.136 | 1.062 | 1.018, 1.108 |

OR = odds ratio; CI = confidence interval; MDM = Northern Ireland Multiple Deprivation Measure income domain; Capital = capital value of housing; Wood = the proportion of woodland; Grass = the proportion of grassland; Q = quintile; G = grade.

Table S2 Sensitivity analysis in the association between green space, SES, and the odds of having a positive COVID-19 test.

|  | Sensitivity analysis 1 | | Sensitivity analysis 2 | | Sensitivity analysis 3 | |
| --- | --- | --- | --- | --- | --- | --- |
|  | OR | 95% CI | OR | 95% CI | OR | 95% CI |
| Male (ref: Female) | 1.179 | 1.165, 1.192 | 1.179 | 1.165, 1.192 | 1.177 | 1.163, 1.191 |
| Age:18-29 (ref: Age: <19) | 0.621 | 0.609, 0.632 | 0.621 | 0.609, 0.632 | 0.629 | 0.617, 0.641 |
| Age: 30-49 | 0.571 | 0.562, 0.581 | 0.571 | 0.562, 0.581 | 0.577 | 0.567, 0.587 |
| Age: 50-69 | 0.434 | 0.427, 0.442 | 0.435 | 0.427, 0.443 | 0.435 | 0.427, 0.443 |
| Age: >69 | 0.287 | 0.279, 0.294 | 0.287 | 0.280, 0.295 | 0.286 | 0.279, 0.294 |
| Urban area (ref: Rural area) | 0.982 | 0.961, 1.003 | 0.978 | 0.958, 0.998 | 0.987 | 0.966, 1.009 |
| MDMQ5 (ref: MDMQ1) | 0.953 | 0.922, 0.986 | 0.894 | 0.867, 0.921 | 0.887 | 0.860, 0.915 |
| MDMQ4 | 0.977 | 0.951, 1.004 | 0.926 | 0.900, 0.952 | 0.924 | 0.897, 0.951 |
| MDMQ3 | 1.007 | 0.980, 1.034 | 0.953 | 0.927, 0.979 | 0.947 | 0.921, 0.974 |
| MDMQ2 | 1.001 | 0.975, 1.027 | 0.983 | 0.957, 1.010 | 0.972 | 0.945, 0.999 |
| CapitalG5 (ref: CapitalG1) | 0.895 | 0.838, 0.955 | 0.899 | 0.842, 0.959 | 0.898 | 0.841, 0.959 |
| CapitalG4 | 1.020 | 0.979, 1.064 | 1.029 | 0.987, 1.073 | 1.020 | 0.978, 1.065 |
| CapitalG3 | 0.998 | 0.977, 1.019 | 1.006 | 0.984, 1.027 | 0.999 | 0.977, 1.021 |
| CapitalG2 | 1.026 | 1.012, 1.041 | 1.032 | 1.018, 1.047 | 1.027 | 1.013, 1.042 |
| WoodQ2 (ref: WoodQ1) | 0.945 | 0.912, 0.980 | 0.967 | 0.937, 0.997 | 0.957 | 0.924, 0.992 |
| WoodQ3 | 0.940 | 0.905, 0.976 | 0.982 | 0.950, 1.016 | 0.953 | 0.918, 0.990 |
| WoodQ4 | 0.962 | 0.922, 1.003 | 0.989 | 0.955, 1.025 | 0.973 | 0.933, 1.014 |
| WoodQ5 | 0.938 | 0.892, 0.987 | 0.959 | 0.924, 0.996 | 0.942 | 0.896, 0.990 |
| GrassQ2 (ref: GrassQ1) | 1.049 | 0.996, 1.104 | 1.021 | 0.987, 1.056 | 1.038 | 0.987, 1.092 |
| GrassQ3 | 1.050 | 1.009, 1.093 | 1.021 | 0.986, 1.057 | 1.046 | 1.005, 1.088 |
| GrassQ4 | 1.065 | 1.021, 1.110 | 1.049 | 1.012, 1.088 | 1.060 | 1.017, 1.105 |
| GrassQ5 | 1.069 | 1.023, 1.116 | 1.032 | 0.995, 1.070 | 1.060 | 1.016, 1.107 |

OR = odds ratio; CI = confidence interval; MDM = Northern Ireland Multiple Deprivation Measure income domain; Capital = capital value of housing; Wood = the proportion of woodland; Grass = the proportion of grassland; Q = quintile; G = grade.

Table S3 Stratified analysis of the association between green space, SES, and the odds of having a positive COVID-19 test.

|  | OR (Males) | 95% CI | OR (Females) | 95% CI | OR (Age: <19) | 95% CI | OR (Age:18-29) | 95% CI | OR (Age: 30-49) | 95% CI | OR (Age: 50-69) | 95% CI | OR (Age: >69) | 95% CI | OR (Urban areas) | 95% CI | OR (Rural areas) | 95% CI |
| --- | --- | --- | --- | --- | --- | --- | --- | --- | --- | --- | --- | --- | --- | --- | --- | --- | --- | --- |
| MDMQ5 (ref: MDMQ1) | 0.905 | 0.870, 0.941 | 0.869 | 0.837, 0.901 | 0.988 | 0.930, 1.049 | 0.860 | 0.816, 0.906 | 0.867 | 0.830, 0.905 | 0.870 | 0.824, 0.919 | 0.799 | 0.725, 0.881 | 0.900 | 0.850, 0.953 | 0.908 | 0.875, 0.942 |
| MDMQ4 | 0.939 | 0.906, 0.974 | 0.906 | 0.876, 0.937 | 0.988 | 0.936, 1.044 | 0.901 | 0.859, 0.944 | 0.901 | 0.866, 0.937 | 0.928 | 0.882, 0.976 | 0.844 | 0.771, 0.925 | 0.947 | 0.904, 0.992 | 0.930 | 0.896, 0.965 |
| MDMQ3 | 0.966 | 0.933, 1.000 | 0.944 | 0.914, 0.975 | 1.009 | 0.958, 1.064 | 0.938 | 0.896, 0.982 | 0.942 | 0.907, 0.978 | 0.960 | 0.914, 1.008 | 0.889 | 0.814, 0.970 | 1.006 | 0.961, 1.054 | 0.938 | 0.907, 0.971 |
| MDMQ2 | 1.000 | 0.966, 1.035 | 0.964 | 0.933, 0.996 | 1.024 | 0.972, 1.080 | 0.942 | 0.901, 0.986 | 0.982 | 0.946, 1.020 | 1.003 | 0.954, 1.054 | 0.880 | 0.807, 0.961 | 1.006 | 0.962, 1.052 | 0.980 | 0.946, 1.016 |
| CapitalG5 (ref: CapitalG1) | 0.844 | 0.772, 0.922 | 0.908 | 0.830, 0.994 | 0.860 | 0.750, 0.985 | 0.876 | 0.766, 1.003 | 0.914 | 0.805, 1.037 | 0.825 | 0.730, 0.932 | 0.720 | 0.556, 0.932 | 0.993 | 0.912, 1.080 | 0.832 | 0.746, 0.928 |
| CapitalG4 | 0.956 | 0.901, 1.015 | 1.065 | 1.005, 1.128 | 1.076 | 0.982, 1.178 | 1.030 | 0.940, 1.128 | 0.964 | 0.890, 1.043 | 0.984 | 0.905, 1.070 | 0.854 | 0.714, 1.020 | 1.061 | 1.008, 1.116 | 0.980 | 0.908, 1.057 |
| CapitalG3 | 0.990 | 0.960, 1.022 | 1.002 | 0.974, 1.032 | 1.024 | 0.976, 1.074 | 1.059 | 1.011, 1.109 | 0.959 | 0.923, 0.995 | 0.989 | 0.946, 1.034 | 0.844 | 0.771, 0.924 | 1.039 | 1.011, 1.068 | 0.948 | 0.915, 0.983 |
| CapitalG2 | 1.038 | 1.017, 1.060 | 1.022 | 1.003, 1.041 | 1.020 | 0.986, 1.055 | 1.078 | 1.046, 1.110 | 1.011 | 0.988, 1.035 | 1.043 | 1.013, 1.074 | 0.956 | 0.907, 1.007 | 1.063 | 1.041, 1.085 | 1.004 | 0.985, 1.023 |
| WoodQ2 (ref: WoodQ1) | 0.940 | 0.903, 0.978 | 0.964 | 0.928, 1.002 | 0.957 | 0.903, 1.014 | 0.940 | 0.894, 0.988 | 0.970 | 0.928, 1.015 | 0.929 | 0.882, 0.979 | 1.016 | 0.929, 1.111 | 0.936 | 0.896, 0.977 | 1.021 | 0.963, 1.081 |
| WoodQ3 | 0.942 | 0.902, 0.983 | 0.950 | 0.911, 0.990 | 1.011 | 0.949, 1.077 | 0.943 | 0.893, 0.996 | 0.973 | 0.927, 1.021 | 0.875 | 0.828, 0.926 | 0.926 | 0.840, 1.021 | 0.896 | 0.852, 0.943 | 1.046 | 0.990, 1.106 |
| WoodQ4 | 0.960 | 0.916, 1.006 | 0.970 | 0.927, 1.016 | 0.978 | 0.912, 1.050 | 0.975 | 0.918, 1.036 | 0.969 | 0.918, 1.022 | 0.936 | 0.879, 0.997 | 0.995 | 0.893, 1.109 | 0.922 | 0.864, 0.985 | 1.052 | 0.993, 1.115 |
| WoodQ5 | 0.942 | 0.890, 0.996 | 0.952 | 0.900, 1.006 | 0.896 | 0.824, 0.976 | 0.961 | 0.895, 1.033 | 0.982 | 0.921, 1.048 | 0.931 | 0.863, 1.005 | 0.984 | 0.861, 1.124 | 0.910 | 0.845, 0.981 | 0.994 | 0.929, 1.064 |
| GrassQ2 (ref: GrassQ1) | 1.002 | 0.946, 1.062 | 1.046 | 0.989, 1.106 | 1.035 | 0.949, 1.129 | 1.034 | 0.961, 1.113 | 1.031 | 0.966, 1.100 | 0.983 | 0.911, 1.061 | 1.021 | 0.895, 1.165 | 0.989 | 0.893, 1.095 | 1.065 | 1.006, 1.127 |
| GrassQ3 | 1.027 | 0.982, 1.074 | 1.044 | 1.000, 1.090 | 1.066 | 0.997, 1.141 | 1.046 | 0.988, 1.107 | 1.036 | 0.986, 1.089 | 1.005 | 0.948, 1.067 | 0.997 | 0.901, 1.103 | 1.121 | 1.035, 1.214 | 1.044 | 0.999, 1.091 |
| GrassQ4 | 1.030 | 0.983, 1.080 | 1.073 | 1.026, 1.123 | 1.035 | 0.965, 1.111 | 1.080 | 1.017, 1.146 | 1.069 | 1.014, 1.126 | 1.029 | 0.967, 1.094 | 1.019 | 0.916, 1.134 | 1.149 | 1.060, 1.246 | 1.008 | 0.961, 1.058 |
| GrassQ5 | 1.029 | 0.980, 1.080 | 1.068 | 1.019, 1.119 | 1.041 | 0.967, 1.120 | 1.092 | 1.026, 1.162 | 1.043 | 0.987, 1.101 | 1.039 | 0.975, 1.108 | 0.967 | 0.866, 1.081 | 1.152 | 1.062, 1.249 | 1.012 | 0.960, 1.066 |

Models were controlled for sex, age, and urbanity. OR = odds ratio; CI = confidence interval; MDM = Northern Ireland Multiple Deprivation Measure income domain; Capital = capital value of housing; Wood = the proportion of woodland; Grass = the proportion of grassland; Q = quintile; G = grade.

Table S4 The association between green space and the odds of having a positive COVID-19 test (stratified by SES).

|  | OR (CapitaG5) | 95% CI | OR (CapitalG4) | 95% CI | OR (CapitalG3) | 95% CI | OR (CapitaG2) | 95% CI | OR (CapitalG1) | 95% CI | OR (MDMQ5) | 95% CI | OR (MDMQ4) | 95% CI | OR (MDMQ3) | 95% CI | OR (MDMQ2) | 95% CI | OR (MDMQ1) | 95% CI |
| --- | --- | --- | --- | --- | --- | --- | --- | --- | --- | --- | --- | --- | --- | --- | --- | --- | --- | --- | --- | --- |
| WoodQ2 (ref: WoodQ1) | 0.926 | 0.555, 1.545 | 0.989 | 0.929, 1.053 | 0.873 | 0.772, 0.988 | 0.966 | 0.920, 1.014 | 0.939 | 0.898, 0.981 | 0.941 | 0.870, 1.019 | 1.026 | 0.934, 1.127 | 0.99 | 0.923, 1.062 | 0.916 | 0.846, 0.991 | 0.937 | 0.872, 1.007 |
| WoodQ3 | 1.009 | 0.608, 1.677 | 0.929 | 0.864, 0.999 | 0.912 | 0.789, 1.054 | 0.936 | 0.889, 0.986 | 0.956 | 0.911, 1.003 | 0.932 | 0.856, 1.014 | 1.048 | 0.952, 1.153 | 0.945 | 0.876, 1.020 | 0.952 | 0.879, 1.031 | 0.953 | 0.881, 1.031 |
| WoodQ4 | 1.092 | 0.658, 1.814 | 0.995 | 0.907, 1.092 | 1.041 | 0.857, 1.265 | 0.941 | 0.889, 0.995 | 0.967 | 0.916, 1.021 | 0.978 | 0.889, 1.076 | 1.09 | 0.985, 1.205 | 0.918 | 0.843, 1.000 | 1.005 | 0.921, 1.097 | 0.909 | 0.834, 0.991 |
| WoodQ5 | 0.908 | 0.550, 1.500 | 0.959 | 0.856, 1.074 | 0.849 | 0.652, 1.106 | 0.959 | 0.897, 1.026 | 0.928 | 0.866, 0.994 | 0.978 | 0.881, 1.086 | 0.914 | 0.809, 1.033 | 0.935 | 0.843, 1.037 | 0.949 | 0.850, 1.060 | 0.895 | 0.802, 0.998 |
| GrassQ2 (ref: GrassQ1) | 0.925 | 0.540, 1.586 | 0.932 | 0.813, 1.068 | 1.326 | 0.973, 1.809 | 0.976 | 0.915, 1.041 | 1.079 | 1.005, 1.158 | 1.05 | 0.952, 1.158 | 1.215 | 1.077, 1.369 | 0.956 | 0.863, 1.059 | 0.977 | 0.871, 1.096 | 1.11 | 0.987, 1.249 |
| GrassQ3 | 1.314 | 0.774, 2.231 | 1.008 | 0.915, 1.110 | 1.288 | 1.048, 1.583 | 1.004 | 0.955, 1.056 | 1.076 | 1.022, 1.133 | 0.989 | 0.909, 1.075 | 1.075 | 0.984, 1.174 | 1.095 | 1.011, 1.186 | 1.051 | 0.964, 1.146 | 1.143 | 1.043, 1.253 |
| GrassQ4 | 1.022 | 0.582, 1.793 | 1.01 | 0.915, 1.115 | 1.237 | 1.005, 1.523 | 1 | 0.947, 1.055 | 1.125 | 1.066, 1.187 | 0.986 | 0.900, 1.079 | 1.081 | 0.991, 1.179 | 1.008 | 0.927, 1.096 | 1.08 | 0.985, 1.183 | 1.176 | 1.070, 1.293 |
| GrassQ5 | 1.168 | 0.664, 2.054 | 1.009 | 0.912, 1.116 | 1.195 | 0.970, 1.473 | 0.993 | 0.939, 1.051 | 1.116 | 1.054, 1.181 | 1.009 | 0.920, 1.106 | 1.042 | 0.944, 1.151 | 0.983 | 0.902, 1.072 | 1.159 | 1.058, 1.270 | 1.181 | 1.071, 1.302 |

Models were controlled for sex, age, and urbanity. OR = odds ratio; CI = confidence interval; MDM = Northern Ireland Multiple Deprivation Measure income domain; Capital = capital value of housing; Wood = the proportion of woodland; Grass = the proportion of grassland; Q = quintile; G = grade.

Table S5 Effect modifications of demographics in the association between SES and the odds of having a positive COVID-19 test.

|  | P_interaction_ | | |
| --- | --- | --- | --- |
| Variable | Sex | Age | Urbanity |
| MDMQ5 (ref: MDMQ1) | 0.020 | 0.000 | 0.745 |
| MDMQ4 | 0.012 | 0.000 | 0.317 |
| MDMQ3 | 0.114 | 0.000 | 0.031 |
| MDMQ2 | 0.008 | 0.000 | 0.535 |
| CapitalG5 (ref: CapitalG1) | 0.234 | 0.068 | 0.039 |
| CapitalG4 | 0.010 | 0.001 | 0.042 |
| CapitalG3 | 0.609 | 0.000 | 0.000 |
| CapitalG2 | 0.051 | 0.000 | 0.000 |
